# PDCM Finder: an open global research platform for patient-derived cancer models

**DOI:** 10.1093/nar/gkac1021

**Published:** 2022-11-18

**Authors:** Zinaida Perova, Mauricio Martinez, Tushar Mandloi, Federico Lopez Gomez, Csaba Halmagyi, Alex Follette, Jeremy Mason, Steven Newhauser, Dale A Begley, Debra M Krupke, Carol Bult, Helen Parkinson, Tudor Groza

**Affiliations:** European Molecular Biology Laboratory - European Bioinformatics Institute, Wellcome Genome Campus, Hinxton, Cambridge CB10 1SD, UK; European Molecular Biology Laboratory - European Bioinformatics Institute, Wellcome Genome Campus, Hinxton, Cambridge CB10 1SD, UK; European Molecular Biology Laboratory - European Bioinformatics Institute, Wellcome Genome Campus, Hinxton, Cambridge CB10 1SD, UK; European Molecular Biology Laboratory - European Bioinformatics Institute, Wellcome Genome Campus, Hinxton, Cambridge CB10 1SD, UK; European Molecular Biology Laboratory - European Bioinformatics Institute, Wellcome Genome Campus, Hinxton, Cambridge CB10 1SD, UK; European Molecular Biology Laboratory - European Bioinformatics Institute, Wellcome Genome Campus, Hinxton, Cambridge CB10 1SD, UK; European Molecular Biology Laboratory - European Bioinformatics Institute, Wellcome Genome Campus, Hinxton, Cambridge CB10 1SD, UK; The Jackson Laboratory, 600 Main Street, Bar Harbor, ME 04609, USA; The Jackson Laboratory, 600 Main Street, Bar Harbor, ME 04609, USA; The Jackson Laboratory, 600 Main Street, Bar Harbor, ME 04609, USA; The Jackson Laboratory, 600 Main Street, Bar Harbor, ME 04609, USA; European Molecular Biology Laboratory - European Bioinformatics Institute, Wellcome Genome Campus, Hinxton, Cambridge CB10 1SD, UK; European Molecular Biology Laboratory - European Bioinformatics Institute, Wellcome Genome Campus, Hinxton, Cambridge CB10 1SD, UK

## Abstract

PDCM Finder (www.cancermodels.org) is a cancer research platform that aggregates clinical, genomic and functional data from patient-derived xenografts, organoids and cell lines. It was launched in April 2022 as a successor of the PDX Finder portal, which focused solely on patient-derived xenograft models. Currently the portal has over 6200 models across 13 cancer types, including rare paediatric models (17%) and models from minority ethnic backgrounds (33%), making it the largest free to consumer and open access resource of this kind. The PDCM Finder standardises, harmonises and integrates the complex and diverse data associated with PDCMs for the cancer community and displays over 90 million data points across a variety of data types (clinical metadata, molecular and treatment-based). PDCM data is FAIR and underpins the generation and testing of new hypotheses in cancer mechanisms and personalised medicine development.

## INTRODUCTION

**Figure 1.**
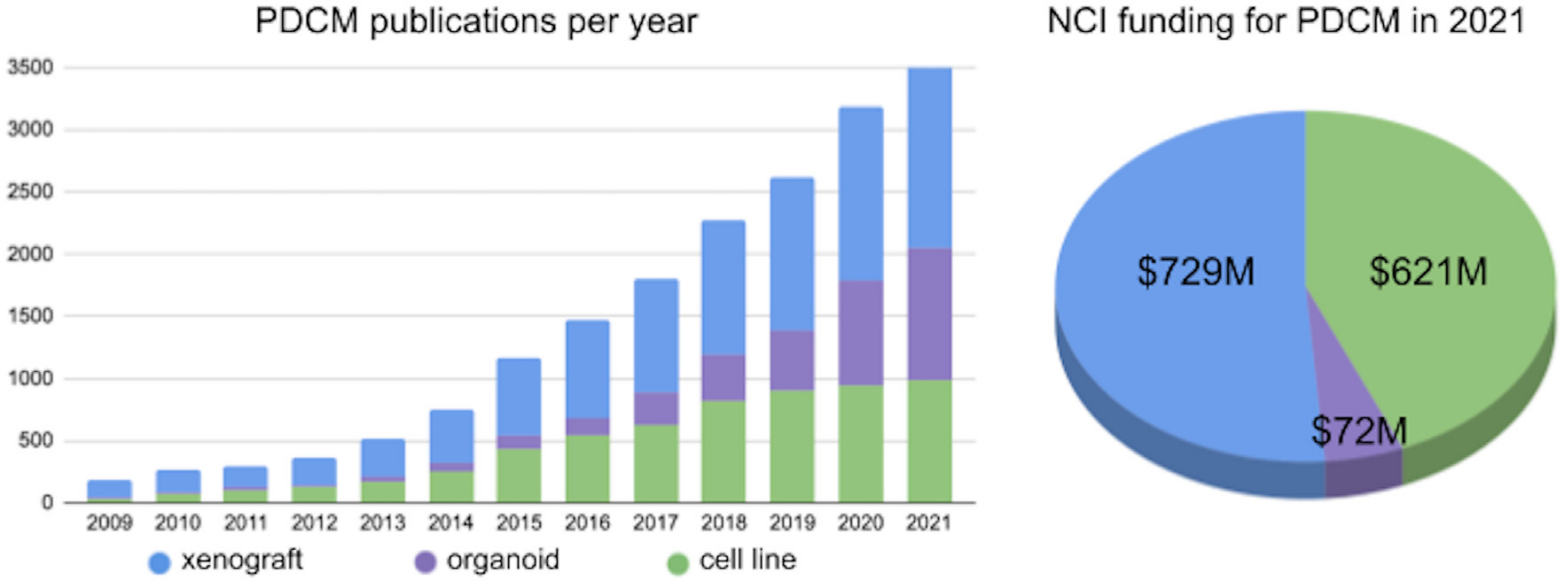
PDCM landscape in 2021. Publications of studies using PDCMs (patient-derived xenograft, organoids or cell lines) have been steadily increasing over the years. NCI funding in 2021 allocated to projects mentioning patient-derived xenografts, organoids or cell lines. Data from Pubmed and NIH RePORTER with the following search queries: xenograft — (patient-derived xenograft) OR (PDX); organoid — (patient-derived organoid) OR (human cancer organoids); cell line — (patient-derived cell line).

Patient-derived cancer models (PDCMs) have become essential tools in both cancer research and preclinical studies and academic and commercial organisations have invested significantly in the generation and characterisation of these models; for example, in 2021 NCI spent ∼685 million USD in active grants that cite PDCMs as part of their activities, and the number of publications using PDCMs has grown exponentially in the last five years (https://reporter.nih.gov/, Figure 1). Each model type has certain advantages and is better suited for specific research areas: cell lines allow high throughput drug screening, organoids model the impact of intratumour heterogeneity, tumour evolution and drug response and PDXs retain the tumour architecture to better predict patient response to treatment (1). As these models gain much of their value through reuse and integration, there is a compelling need for PDCM datasets to adhere to the FAIR data principles (2) — i.e. to be findable, reusable, interoperable and reusable.

PDCM stakeholders, from basic and clinical researchers to bioinformaticians and tool developers, currently navigate a complex landscape to find PDCMs and associated data across multiple commercial and academic resources without being able to rely on shared data standards or interoperable data. PDX Finder has successfully addressed this challenge for the PDX community by standardising and integrating over 90 million data points from >4500 PDX models. The Patient-Derived Cancer Model Finder (PDCM Finder) extends the previous scope by aggregating, standardising and harmonising 6360 models and associated data from PDCM providers, including individual research laboratories, large consortia and contract research organisations. The data model for PDCM Finder extends the minimal information standard for PDX models (PDX-MI) developed in collaboration with a broad range of stakeholders who produce and/or use PDCMs in basic and/or preclinical research (3). Molecular data associated with the models is often obtained using different technologies and presents interoperability challenges in grouping and combining models across platforms and producers. We also adhere to existing standards, such as GA4GH standards for variant representation, genome build, amino acid change, mutation consequence, as well as use nomenclature and ontologies to enable integration (https://www.ga4gh.org/genomic-data-toolkit/). The resource therefore provides a unified entry point for research and clinical communities to search and compare PDCMs and their associated data including frequently mutated genes, diagnoses, drug treatments and sequence data. As the landscape of PDCM models is constantly evolving and new models are both generated and analysed continuously, we recognise the necessity of refreshing PDCM datasets in a timely manner. By using automated validation, clean-up and mapping and hence, minimising the time it takes to process the data, PDCM Finder aims to move from the quarterly release schedule to a release-as-you-upload schedule.

## DATA STANDARDISATION AND AGGREGATION

PDCMs are an invaluable oncology research platform to study cancer progression, mechanisms of drug resistance and predicting response to anti-cancer therapeutic compounds. The heterogeneity of the underlying metadata and the lack of robust standards to describe and publish PDCMs make it difficult for researchers to find models of interest and compare associated data across multiple academic and commercial sources. For example, model providers might use different terms for the same cancer diagnosis, such as ‘breast cancer’ versus ‘breast malignant neoplasm’, or implement different methods for variant analysis. In addition, deposition of molecular data generated from PDCMs and the metadata required for reanalysis has been poor. In 2017, PDX-MI was adopted by the cancer community, including academic entities (EurOPDX consortium, https://www.europdx.eu/) and commercial databases (Repositive, https://repositive.io/; Charles River Tumour Model Compendium, https://compendium.criver.com/). Moreover, European Nucleotide Archive (ENA, https://www.ebi.ac.uk/ena/browser/view/ERC000051) uses PDX-MI as a checklist for sequence file deposition.

PDX-MI provides a standardised format for sharing information about PDX models. It consists of four modules that describe the generation and validation of a PDX model. The Clinical module captures information about the patient and tumour (age, sex, ethnicity and diagnosis and tumour classification, anatomical location, histopathology, specific diagnostic markers). The Model Creation module records characteristics relevant to creating PDX models (e.g. host strain information, the engraftment type and the implantation site). The Model Quality Assurance module consists of attributes related to tissue provenance and fidelity of the passaged tumour, and validation technique(s). Finally, the Model Study/Associated Metadata module includes information about the genomic characterisation and/or treatment in controlled drug dosing studies, and any other additional metadata, such as accession IDs and publications. The attributes within each module are either ‘essential’ — required for accurate description of the PDX model or ‘desirable’ — frequently recorded by the model providers and useful to researchers.

PDX-MI has pioneered the adoption of standards in the PDX community. It has, however, also highlighted the necessity of a minimal information standard for other PDCMs, such as organoids and cell lines. From a standardisation perspective, most attributes defined by PDX-MI can be adopted to describe other PDCMs. For example, all patient-related and tumour-related attributes in the Clinical module are relevant irrespective of the model type. Initial work on developing a generic PDCM standard was done by analysing the internal standards of Cell Model Passports portal (4) and NCI HCMI Searchable catalog (https://hcmi-searchable-catalog.nci.nih.gov/), which resulted in a set of model type specific fields, such as growth properties, sampling day and model relation. In parallel, we started collecting feedback from the community on essential and desirable attributes for other types of PDCMs with the aim to create and publish a new standard early next year. We will apply it to the current 1655 organoid and cell line models in the PDCM Finder and create corresponding facets in the portal. This will enable users to perform model-type specific filtering for organoid and cell line models.

The use of community developed and adopted terminologies underpins the standardisation and integration efforts of the resource. Cancer type, diagnosis and treatments, including names of the drugs, compounds and regimens are reused from NCI Thesaurus (5), human gene names and symbols from HUGO Gene Nomenclature Committee (6) and host strain nomenclature follows the official guidelines from the International Committee on Standardized Genetic Nomenclature for Mice (7).

Currently, PDCM Finder hosts 6316 model entries, and extends the original scope of PDX Finder (8) to include organoids and cell lines. More concretely, it includes 4661 xenograft, 1547 cell line and 108 organoid models from 27 providers, all standardised to the current PDX-MI. The PDX models and their associated data have been migrated from the PDX Finder, and organoid and cell line models were integrated from the Cell Model Passports and the NCI HCMI Searchable catalog.

In addition to model metadata, PDCM Finder supports the following data types: gene expression, gene mutation, copy number alteration (CNA), cytogenetics, patient treatment and drug response. The resource provides molecular data summaries for PDX models including gene mutation (49% of all models from 13 sources), copy number alteration (CNA, 35% of all models from 10 sources), transcriptomics (30% of all models from 7 sources), drug dosing (10% from 4 sources), patient treatment (4% from 6 sources) and cytogenetics (3% of all models from 10 sources). These data are available for download for further analysis from the Model details page in the Web portal. All metadata and data are also accessible via Application programmatic interface (API, documentation available at https://documenter.getpostman.com/view/979205/UzJESJjr).

## USE CASES

Generation and characterisation of PDCMs is an area of significant growth in cancer research. However, these models gain much of their value through reuse and integration — specifically, running aligned experiments with different types of models of the same cancer enables testing new hypotheses. In addition, PDCM data is highly desirable for its translatability to clinical outcomes and many stakeholder needs can be met when PDCM datasets adhere to the FAIR data principles, as presented in detail in Table 1. By improving the FAIRness of these models, PDCM Finder facilitates their use and reuse and underpins new discoveries in a wide variety of cancer research programs.

**Table 1.** Making PDCM data FAIR addresses many use cases for various PDCM stakeholders. For a basic researcher PDCM Finder allows to find and compare models with specific oncogenic mutation from multiple model providers and contact provider about obtaining the chosen model, and submit their study data generated from the model to the resource

Stakeholder	Findable	Accessible	Interoperable	Reusable
Basic researcher	PDCM that carries an oncogenic mutation	Where to obtain PDCM	Be able to group models by oncogenic mutation	Report their study data so can be reused
Translational researcher	PDCM that matches a patient diagnosis and ethnicity	Where to obtain PDCM	Clear protocols on drug dosing, how response was measured	A place to deposit their data to benefit others
Clinical researcher	PDCM drug response dataset that informs clinical decision making	Speedy access to drug response summaries	Find data by drug synonyms, equivalence dosing from model to patient	Report how well patient response predicted by model
Bioinformatician	Datasets by model type, diagnosis, drug response, etc.	Where is data and if controlled or limited access	Harmonised datasets to perform machine learning analysis	Repeat analysis with new data. Allow others to extend analysis
Editor	Necessary data to describe PDCM is provided	Clear links to where PDCM data can be obtained	Study is comparable to other published studies	Allow others to replicate results from study
Integrating tools	Find standardised connection points between datasets	Stable API to access data	Minimise mappings that need to be performed	Be able to add new data sets with each release of tool
Funders	Maximise exposure to funder resources	Increase impact of funded resources	Synergies with other funded efforts	Resources live beyond funding cycle

## PDCM FINDER PORTAL

**Figure 2.**
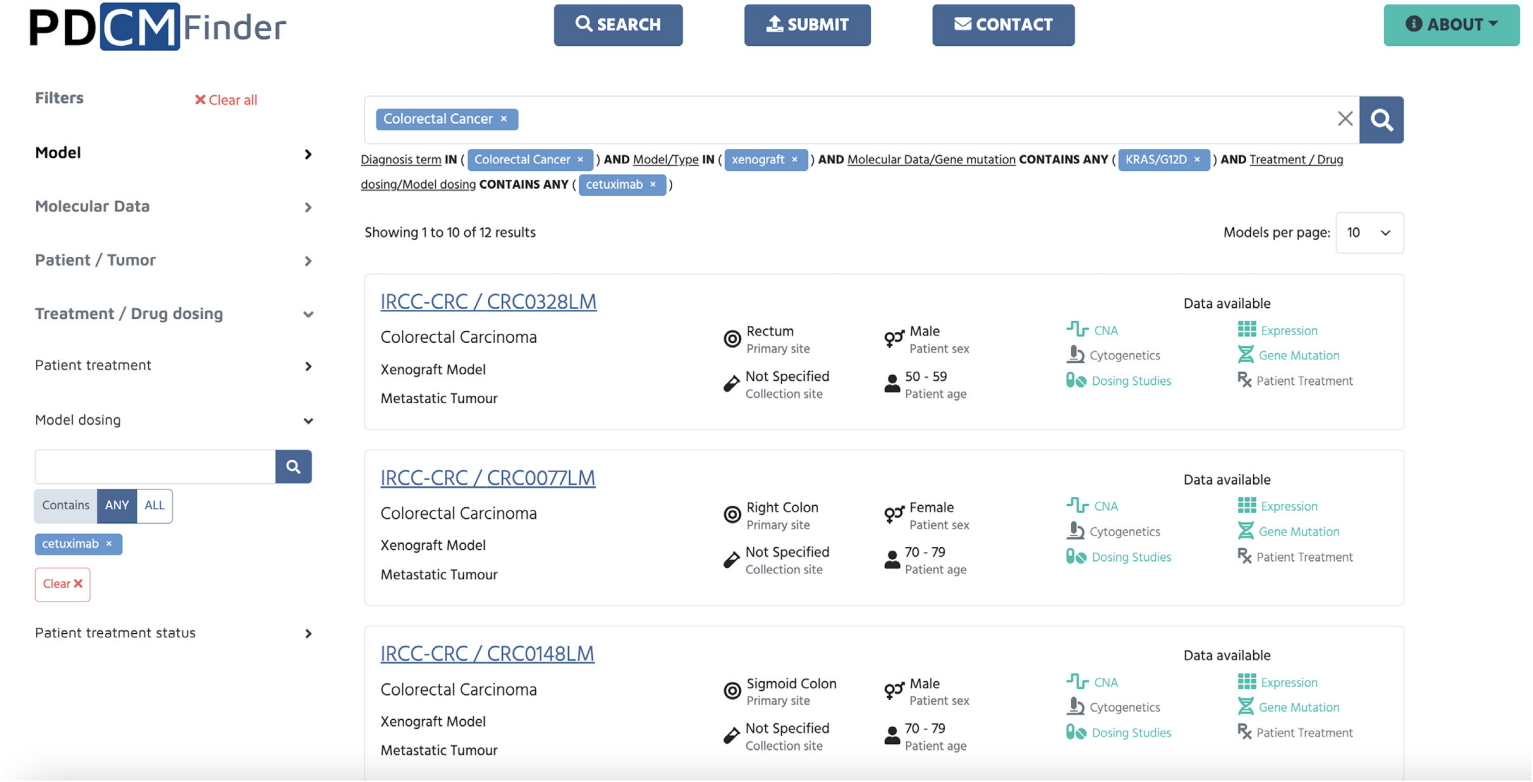
Search results for colorectal cancer xenograft models with KRAS/G12D mutation and cetuximab drug dosing study. Filters are grouped in categories on the left, and selected filters are shown under the search bar. Filters can be reset individually below the search bar or cleared all together by ‘Clear all’ button. Results are presented in a card view with tumour and patient metadata and clearly indicated available data for this model in green colour.

There are several points of entry to explore the data in the PDCM Finder. The Data overview section presents interactive visualisations of ‘frequently mutated genes’, ‘dataset availability’ and ‘top used drug treatments’. The user can search for models based on the cancer diagnosis or by using specific filters in the Search page. Filters are grouped by categories in accordance with published minimal information standards and can be selected by expanding a facet and further selecting one or more filters in the relevant sub-categories. For example, the user can look up ‘colorectal cancer’ using the search bar on the landing page and filter for Type in the Model category to explore 1312 xenografts, 81 cell line and 47 organoid models of colorectal cancer. Results can be further refined by gene mutation, for example ‘KRAS/G12D’, and filtered for model dosing ‘cetuximab’ (Figure 2). Filters can be individually removed or added to adjust the search criteria. Results are presented as cards so that the user can easily scan through attributes such as model type and tumour type, primary site and collection site, patient's age and sex. Coloured icons indicate which data is available for the models.

**Figure 3.**
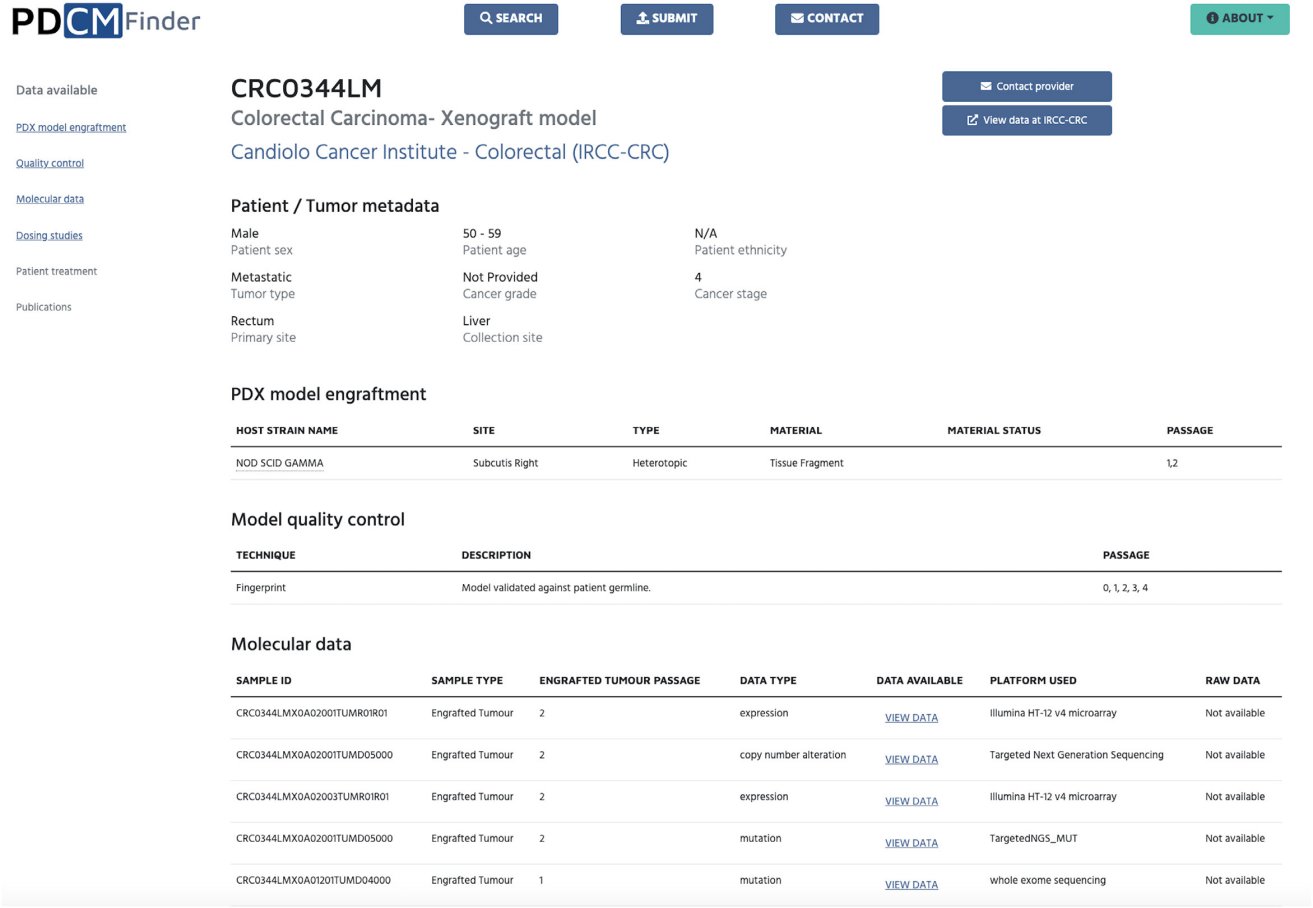
Model details page for the colorectal cancer xenograft model CRC0344LM. In the top section, there is the model ID, followed by the cancer diagnosis and model type, and provider. Next sections include patient and tumour metadata, model generation details (PDX model engraftment in this example), model quality control, molecular data and dosing study results (not shown in this figure). The navigation bar on the left allows the user to move between the sections.

To see further information on the model of interest the user should click on the Model ID which opens Model details page (Figure 3). It presents further model and patient metadata, available molecular and treatment data and associated publications. This allows the user to get an overall assessment of the richness and suitability of the model, as well as explore available data in detail. For example, the user can do a quick check of a specific gene(s) mutation consequence or a change in expression of the gene of interest for this model directly in the browser. If a more thorough comparison is needed, the data can be downloaded for offline analysis. In addition, this view enables the user to contact the provider to request this model or view the data at the provider's webpage. Links to available raw data and descriptions of the platforms used to obtain the data are also available in the Model details page.

As shown, users can find, group and locate PDCMs of all types based on community-defined attributes (e.g. diagnosis, oncogenic mutation, biomarker), explore and download molecular data summaries and drug response data, aggregate and further analyse harmonised PDCM molecular datasets (for example, on cloud-based analysis platforms). PDCM Finder accelerates cancer research by allowing clinicians and researchers to find PDCM data that best matches their patients and/or research questions and explore new therapeutic avenues for patients.

The resource is distinct from other similar initiatives by having a greater breadth and detail of models, aggregating models from both academic and commercial suppliers and being free at the point of data access. This contrasts with resources that only provide access to data they distribute (e.g. Charles River Tumour Model Compendium, https://compendium.criver.com/), academic consortia focused on generating and analysing data, such as PDXNet (https://www.pdxnetwork.org/) and EurOPDX (https://www.europdx.eu/) or commercial entities charging customers a fee to find models. Within the HCMI Initiative there are several portals displaying models generated by the same project, however these lack shared standards, hence making it difficult for researchers to navigate them and implicitly diminishing the value of the produced PDCMs. PDCM Finder enables its users to maximise the impact of their work by removing the barriers to data sharing. It addresses the challenges many users face - searching for models over many repositories implemented using incompatible standards that make analysis and reuse of models difficult, and looking for molecular datasets annotated with insufficient information, which prevent cloud-based analysis. The resource is also unique within the ecosystem of preclinical model resources as it aggregates PDCM data and makes it FAIR while providing clear attribution to the originating resource. The users can search harmonised data across multiple sources and choose to access the data either via PDCM Finder or to directly contact the provider via the link provided in the Model details page.

## IMPLEMENTATION

**Figure 4.**
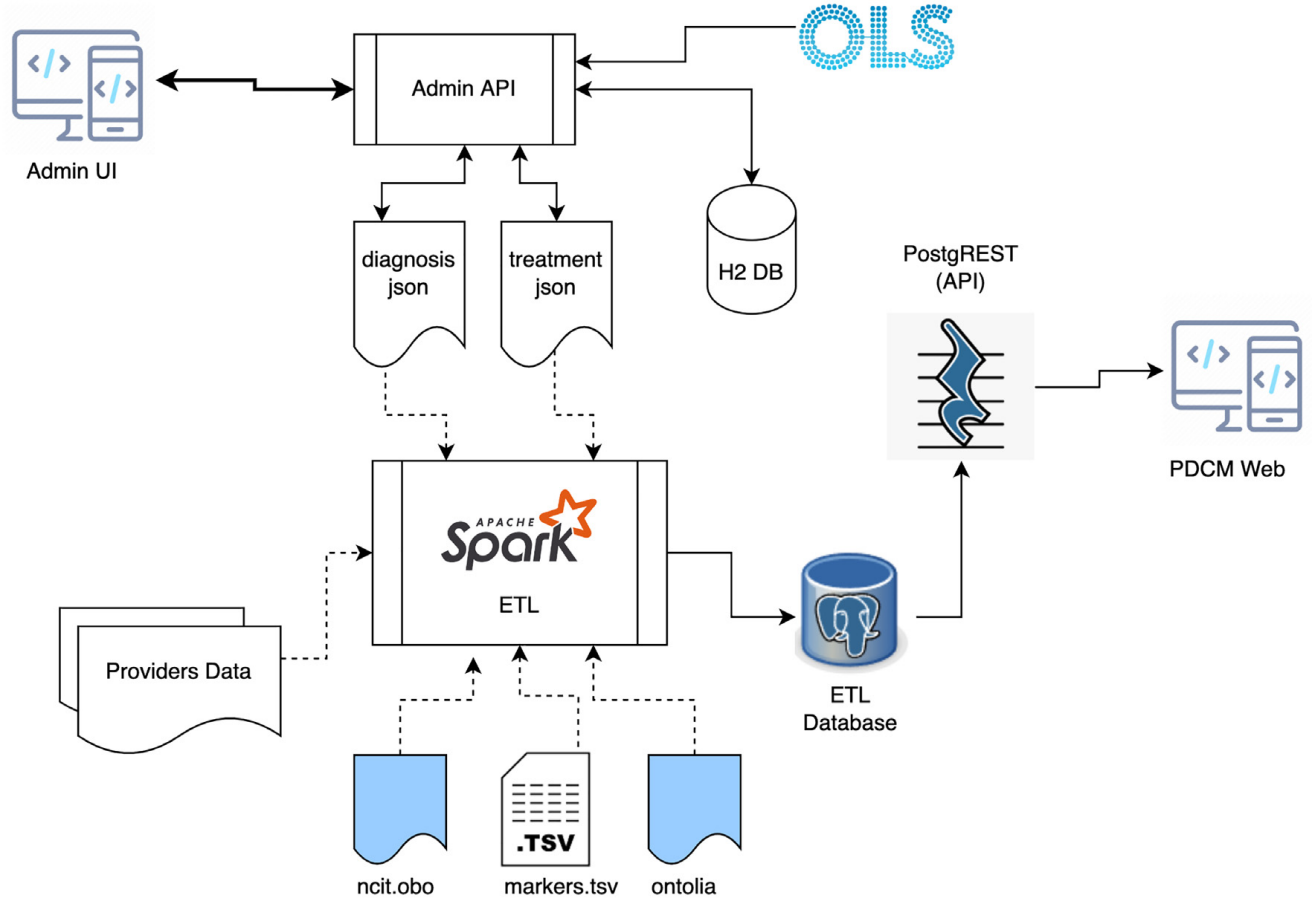
PDCM Finder microservices architecture. Extraction, Transformation and Loading (ETL) pipeline is the central point of all the information processing. It extracts Providers Data from metadata and data files that providers submit about their models, harmonises it according to loaded ontologies and mapping rules (ncit.obo, markers.tsv, ontolia, diagnosis.json, treatment.json), transforms it to be suitable for storage in a relational database and loads results into a PostgreSQL database (ETL Database). PostgREST exposes the content of the ETL database as a REST API and powers up the PDCM Finder Web portal. Admin UI is a web application that allows to create mappings between raw data from diagnosis/treatments and ontology terms from the Ontology Lookup Service (OLS) at the EBI. Created mapping rules are stored in the H2 database and can be easily updated if needed.

PDCM Finder is built using a microservices architecture (Figure 4). This approach enables an independent lifecycle for the individual components, in addition to improving the reusability of our software. The resource uses a new PostgreSQL database and a new database schema, which has increased the efficiency of querying for genomic data and provides us with the flexibility to add additional attributes for new models.

The new architecture also includes universal templates for the new type of models and data (available to download from https://www.cancermodels.org/submit), a custom Extraction, Transformation and Loading (ETL) pipeline using an industry-standard analytics engine (Apache Spark, https://spark.apache.org/) for integration, harmonisation and mapping and a comprehensive API used by an updated frontend built with ReactJS and TypeScript. We use Ontology Lookup Service (OLS,^9^) at the EBI to retrieve ontology terms during the mapping process.

Molecular datasets are routinely generated in validation and use of the PDCMs, however their deposition in data repositories with the metadata necessary to find and use them remains poor. Expanding the resource to new model types requires us to collect data in new and improved ways. In addition to aggregation, PDCM Finder is significantly invested into data cleaning, curation, validation, standardisation and harmonisation via automated means, to support the community's increased adherence to these standards. These efforts will facilitate integration of data across model types at scale enabling many different types of new studies, such as machine learning analysis of harmonised data. All tools and pipelines developed in the context of the platform are freely available for reuse via GitHub: https://github.com/PDXFinder.

## DISCUSSION AND FUTURE DIRECTIONS

Availability and FAIRness of PDCMs and associated data is a bottleneck for efficient hypothesis testing in tumour biology research and new treatment discovery. PDCM Finder aggregates, integrates and presents PDCM information from 27 academic and commercial providers, and provides molecular data summaries to help researchers find their model(s) of interest. It plays an essential role in the cancer community by reducing barriers to data sharing in the constantly evolving landscape of PDCMs. Our primary goal in the near future is to increase the number of PDCMs represented in PDCM Finder by uploading xenograft, organoid and cell line models from existing providers and contacting individuals and organisations that maintain PDCM repositories.

Submission of data to various archives and repositories takes time and is a barrier to data sharing. We are collaborating with the recently launched PDXNet Portal (10) that centralises access to the models generated by the NCI funded PDXNet Consortium, to harmonise the data annotations and streamline the model and data ingestion between the PDXNet Portal and PDCM Finder. PDXNet centres have been submitting their models and data to PDCM Finder individually as any other PDCM provider. We are working on a solution such that upon submitting the model and supporting data to the PDXNet Portal, this will become immediately available also in the PDCM Finder, and interoperable with the rest of the PDCM Finder collection. This approach will remove barriers to data sharing and make it more efficient, and will be expanded to other repositories for all depositors of PDCMs to benefit. By aggregating and integrating PDCM datasets we ensure interoperability and reusability of data, including on emerging cloud platforms, such as NCI Cancer Research Data Commons (https://datacommons.cancer.gov/). PDCM Finder will significantly reduce data deposition complexity by defining a standard format for sharing PDCMs and associated data, externalising our validation processes and implementing new processes for updates. These new services will be made publicly available with full documentation, as well as training materials, and will promote improved data flow across the resource ecosystem.

We encourage model providers to submit genomic datasets and drug dosing studies associated with their models to enhance the value of information we make discoverable to end users through PDCM Finder. We initiated coordinating activities with existing molecular archives to deposit data generated from PDCMs and piloted deposition of raw sequence files to European Nucleotide Archive (11). We plan to provide links to other archives where raw data files associated with the PDCMs are deposited, such as European Genome-phenome Archive (EGA,^12^) and NCBI’s Database of Genotypes and Phenotypes (dbGAP,^13^) in case of controlled access. We will also coordinate submission of sample metadata to BioSamples (14), or BioSample database (15), which provide unique identifiers linking various types of data from the same sample in EBI or NCBI archives, respectfully.

Data quality and provenance are significant issues in an environment where the speed of data generation surpasses the speed at which data are processed and made available. The scientific community will greatly benefit from quality standards for both biological samples and data that are community-driven, enforced by organisations at the forefront of scientific research and are supported by the institutions and journals. Some efforts are in place to achieve this, such as the Standards Initiative by the International Society for Stem Cell Research (ISSCR, https://www.isscr.org/standards). In the absence of community-adopted standards, data generators should maximise efforts to retain and report all available metadata and provenance during the generation of data and its deposition to archives, and data consumers should check the quality of public data prior to using it for their needs.

PDCM Finder requires model providers to include the quality assurance/quality control information for their models during submission process. This information can be found in the Model Quality Assurance section of the PDCM Finder portal. The main goal of PDCM Finder is to provide users with enough information about the models to enable comparison of models from multiple repositories. PDCM Finder does this through aggregation, standardisation and harmonisation of model metadata and data, empowering users to make a choice suitable for their specific needs and requirements.

PDCM Finder follows user-centered development, and the next features will be determined by the needs of PDCM community. We will continue to assess user needs by surveys and user testing of the PDCM Finder, as well as stay up to date with the current PDCM landscape and other cancer informatics resources. We are committed to collaborative development and reuse of the informatics tools, and will evaluate the existing software developed by other groups when planning implementation of new functionality in the PDCM Finder. In the first instance we will integrate with several cancer annotation resources, including those funded by the NCI’s Information Technology for Cancer Research (ITCR) program (https://itcr.cancer.gov/), such as CiViC (https://civicdb.org/), OnkoMX (https://www.oncomx.org/), OpenCravat (https://opencravat.org/) and Wellcome Trust funded COSMIC (https://cancer.sanger.ac.uk/cosmic). We will continue to work with related PDCM initiatives, such as PDXNet, PDMR (https://pdmr.cancer.gov/), EurOPDX and continue developing software components usable by PDCM-focused and other projects in the model ecosystem (for example, EurOPDX Data Portal,^16^). As the size and complexity of the PDCM datasets grow we will extend PDCM Finder capabilities and continue to improve its value. We will do so by expanding the coverage of data available in the public domain and ensuring the data are better integrated into the data ecosystem.

## DATA AVAILABILITY

PDCM Finder is an open project available in the GitHub repository (https://github.com/PDCMFinder) under Apache 2.0 license (https://www.apache.org/licenses/LICENSE-2.0). Model metadata and associated data is available to download from the portal website or via API under the general EMBL-EBI terms of use (https://www.ebi.ac.uk/about/terms-of-use).

## Notes

Present address: Alex Follette, Clinical Bioinformatics Unit, Victorian Clinical Genetics Services, Parkville, Victoria 3052, Australia.

Present address: Jeremy Mason, Data Science, IMU, London SE1 1UL, UK.
